# Does time-to-surgery affect mortality in patients with acute osteoporotic vertebral compression fractures?

**DOI:** 10.1186/s12877-021-02682-0

**Published:** 2021-12-18

**Authors:** Christian Pfeifle, Petr Kohut, Jan-Sven Jarvers, Ulrich J. Spiegl, Christoph-Eckhard Heyde, Georg Osterhoff

**Affiliations:** Department of Orthopaedics, Trauma and Plastic Surgery, University Hospital Leipzig, 04103 Leipzig, Germany

**Keywords:** Geriatric, Osteoporosis, Mortality, Vertebral compression fracture, Complications, Fracture

## Abstract

**Introduction:**

Osteoporotic vertebral compression fractures (VCFs) are common. An increase in mortality associated with osteoporotic VCFs has been well documented. The purpose of this study was to assess the impact of time to surgery on 1-year survival in patients with osteoporotic vertebral compression fractures.

**Methods:**

In a retrospective cohort study with prospective mortality follow-up, **c**onsecutive patients aged ≥ 60 years who had operative treatment of a low-energy fracture of a thoracolumbar vertebra and had undergone surgical stabilization between January 2015 and December 2018 were identified from our institutional database. By chart review, additional information on hospitalization time, comorbidities (expressed as ASA - American Society of Anesthesiologists Scale), complications and revision surgery was obtained. Time-to-surgery was defined as the time between admission and surgery. Mortality data was assessed by contacting the patients by phone, mail or the national social insurance database.

**Results:**

Two hundred sixty patients (mean age 78 years, SD 7 years, range, 60 to 93; 172 female) were available for final analysis. Mean follow-up was 40 months (range, 12 to 68 months). Fifty-nine patients (22.7%) had died at final follow-up and 27/260 patients (10.4%) had died within 1 year after the surgery. Time-to-surgery was not different for patients who died within 1 year after the surgery and those who survived (*p* = .501). In-hospital complications were seen in 40/260 (15.4%) patients. Time-to-surgery showed a strong correlation with hospitalization time (Pearson’s *r =* .614, *p* < .001), but only a very weak correlation with the time spent in hospital after the surgery (Pearson’s *r =* .146, *p* = .018).

**Conclusions:**

In contrast to patients with proximal femur factures, time-to-surgery had no significant effect on one-year mortality in geriatric patients with osteoporotic vertebral compression fractures. Treatment decisions for these fractures in the elderly should be individualized.

## Introduction

Vertebral compression fractures (VCFs) are the most common osteoporotic fractures, with an incidence of aproximately 1,4 million VCFs diagnosed annualy throughout the world [1]. Osteoporotic VCFs occur as a result of low energy trauma and can be induced by everyday activities, such as bending forward, lifting objects, and climbing down stairs [1, 2]. Aproximately one in four VCFs are associated with immobilizing pain, which can result in imobility-associated complications and death in geriatric patients [3]. An increase in mortality associated with osteoporotic VCFs has been well documented. Even 5 years after an VCF, a higher mortality than that of the general population can be observed [4]. In some studies, VCF patients who received operative treatment experienced lower mortality and morbidity than patients who recieved conservative management [5–7].

Wait times to surgery have proven to be a significant factor in predicting mortality and complications in geriatric patients with proximal femur fractures [8, 9] and time-to-surgery is being used as quality-of-care indicator worldwide. Hence, surgical intervention for proximal femur fractures in the elderly is generally recommended within 48 h after the injury. A similar reduction in mortality with early operative treatment has been reported for geriatric distal femur fractures [10].

Although time-to-surgery has been linked to mortality following different types of fractures, the relationship between wait time to surgery and mortality in geriatric patients with VCFs has not yet been investigated.

Thus, the purpose of this study was to assess the effect of time-to-surgery on 1-year survival in patients with osteoporotic vertebral compression fractures.

## Materials and methods

### Patients

The study protocol was approved by the institutional ethics committee (reference 202/19-ek). Consecutive patients aged ≥ 60 years who had operative treatment of a low-energy compression fracture of a thoracolumbar vertebra and had undergone surgical stabilization between January 2015 and December 2018 were identified from our institutional database. For all patients identified from this database, a retrospective chart review was performed and eligibility was assessed. Exclusion criteria included fractures due to a high energy trauma, fractures that were treated non-operatively, pathologic fractures, and the presence of additional injuries. Also, patients who had expressed objection to the use of their data for research purposes were excluded.

By chart review, additional information on hospitalization time, comorbidities (expressed as ASA - American Society of Anesthesiologists Scale), complications and revision surgery were obtained. Time-to-surgery was defined as the time between admission and surgery. Mortality data was assessed by contacting the patients by phone. If the patients could not be contacted by a first phone call, two more attempts were made by phone and mail. In case personal contact could not be established, mortality was assessed using the national social insurance database (Deutsche Rentenversicherung) that provides mortality data for every German resident who received statutory pension.

### Outcome

Primary outcome was the all-cause mortality rate 1 year after surgery.

Secondary outcomes were in-hospital complications, duration of the hospitalization and duration of hospitalization after surgery.

### Statistical analysis

Statistical analysis was performed in SPSS 24.0 (SPSS Inc., Chicago, IL, USA). Unless otherwise denoted, data was summarized as mean with standard deviation (SD).

Primary outcome was death within 1 year after surgery. The hypothesis was that time-to-surgery is an independent risk factor for not surviving the first year after surgery. To assess for potential confounders, baseline characteristics were compared between patients who deceased within the first year and those who did not (Table 1). Nominal variables were associated using Chi-Square or Fisher’s exact tests and Student’s T-test was used to compare normally distributed continuous data. Pearson’s *r* was used for correlation of continuous data. Logistic regression analysis was performed to assess the impact of “time-to-surgery” by adjusting for age, fracture pattern (Osteoporotic Fracture [OF] classification [11]) and perioperative risk profile (ASA scale). The level of statistical significance was set at *p* < 0.05.

**Table 1:** Patients’ baseline characteristics (*N* = 260)

	Survived	Deceased^a^	***p***	Total
**N**	233	27		260
**Age [y]**	78, SD 7	77, SD 7	.566^b^	78, SD 7
**Gender [f**: **m]**				172: 88
**ASA**			.008^c^	
1	3	0		3
2	81	1		82
3	148	26		174
4	1	0		1
**OF classification**			.418^c^	
1	4	0		4
2	75	5		80
3	122	17		139
4	32	5		37
**Type of surgery**			.412^c^	
Kyphoplasty	55	6		61
CPPPSF+Kypho	130	14		144
Open posterior fixation	48	7		55

^a^within 1 year after surgery

^b^Student’s T-test

^c^Pearson Chi-square/Fisher’s test

*ASA* ASA - American Society of Anaesthesiologists Scale, *OF classification* Osteoporotic Fracture classification, *CPPPSF + Kypho* cemented percutaneous posterior pedicle screw fixation with kyphoplasty of the fractured vertebra

## Results

Two-hundred and sixty patients (mean age 78 years, SD 7 years, range, 60 to 93; 172 female) were available for final analysis (Table 1). Mean follow-up was 40 months (range, 12 to 68 months). The mean length of hospitalization was 16, SD 10 days (Table 2). The mean time from admission to surgery (“time-to-surgery”) was 7, SD 5 days.

**Table 2:** Time-to-surgery, hospitalization time and time after surgery

	Survived	Deceased^a^	***p***
**N**	233	27	
**Time-to-surgery [d]**	7, 5 SD	8, SD 6	.501^b^
**Hospitalization time [d]**	16, SD 9	18, SD 12	.311^b^
**In-hospital time after surgery [d]**	9, SD 8	10, SD 8	.471^b^

^a^within 1 year after surgery

^b^Student’s T-test

### One-year mortality

Mortality data could be obtained for all patients. Fifty-nine patients (22.7%) had died at final follow-up and 27/260 patients (10.4%) had died within 1 year after the surgery.

Time-to-surgery was not different for patients who died within 1 year after the surgery and those who survived (*p* = .501). This was also the case if only patients with an ASA scale of 2 or more were included into the analysis (*p* = .855). When looking at patients who were operated within 48 h after admission, again, there was no difference in one-year mortality compared to patients who had waited longer for surgery (*p* = .559). In addition, one-year-mortality was not associated with fracture pattern (OF classification, *p* = .418) or type of surgery (*p* = .814).

A logistic regression analysis confirmed no relevant effect of “time-to-surgery” on the risk for death within 1 year even when adjusting for age, fracture pattern and ASA. In contrast, ASA itself was found to be an independent risk factor for death within 1 year after surgery (Odds Ratio 8.959, 95% CI 1.998 to 40.163, *p* = .004).

### Complications

In-hospital complications were seen in 40/260 (15.4%) patients. Complications included infection in 33 (12.7%), thrombo-embolic events in 3 (1.2%), delirium in 4 (1.5%), and in-hospital death in five (1.9%) cases.

There was no difference in time-to-surgery comparing patients with in-hospital complications (8, SD 6 days) and patients without complications (7, SD 5 days, *p* = .198). This was also the case when comparing patients with and without in-hospital infection (7, SD 5 days vs. 7, SD 5 days, *p* = .802), thrombo-embolic events (9, SD 5 days vs. 7, SD 5 days, *p* = .388), delirium (5, SD 2 vs. 7, SD 5, *p* = .464), and in-hospital death (8, SD 8 days vs. 7, SD 5 days, *p* = .696).

Complications were also not associated with fracture pattern (*p* = .077) but were seen more frequently in open surgery (16/55, 29.0%) compared to kyphoplasty (7/61, 11.5%) and percutaneous posterior fixation with kyphoplasty 17/144, 11.8%, *p* = .030). This effect was mainly based on a higher rate of wound infections which was found in 8 of 55 open surgery cases (14.5%) compared to 0 in 61 cases (0%) with kyphoplasty and 3 in 141 (2.1%) with percutaneous posterior fixation (p = < .001).

### Hospitalization time

Time-to-surgery showed a strong correlation with hospitalization time (Pearson’s *r =* .614, *p* < .001), but only a very weak correlation with the time spent in hospital after the surgery (Pearson’s *r =* .146, *p* = .018) [12].

## Discussion

The aim of this study was to evaluate the mortality of patients that had undergone surgical stabilisation of osteoporotic vertebral fractures and to assess the effect of time-to-surgery on mortality, complications and hospitalization time.

In this cohort of geriatric patients with osteoporotic vertebral fractures, all-cause mortality was 10.4% regardless of the time between admission and surgery. In this study, time-to-surgery had no significant effect on one-year mortality. The ASA score showed a significant correlation with one-year mortality, and time-to-surgery did strongly correlate with the duration of the whole hospitalization. In addition, open surgery was associated with a significantly higher rate of wound infections compared to percutaneous methods.

A mortality rate of 10.4% is consistent with previous studies that show mortality rates between 7. % and 14.6 1 year after surgical stabilisation of a vertebral body fracture [13, 14].

Patients who waited several days before surgery had the same mortality and complication rate like patients that were operated during the first 48 h. A direct comparison with literature cannot be made as there are no recent studies examining the mortality rates of patients after spinal stabilisation depending on the time-to-surgery. In contrast to our study the advantage of early surgery in patients with proximal femur fractures is widely known [15].

We found significantly lower rates of wound infections in patients who had undergone percutaneous posterior stabilisation compared to those who had undergone open surgery. This is in line with previous studies on this topic that show fewer surgical site infections in percutaneous procedures [16].

As time-to-surgery showed a strong correlation to the overall hospitalisation time but not to the time spent in hospital after surgery, we conclude that there is no significant difference in recovery time depending on time-to-surgery. However, most patients with long time-to-surgery were mobilized before surgery because non-operative treatment was endeavored initially and patients with comorbidities may have needed some days of preoperative conditioning. The indication to surgery in these cases was made based on radiological fracture progression or persisting strong pain.

The limitations of this study are inherent with its retrospective design. Therefore, selection bias for operative treatment is possible, since less painful fractures with a less complex morphology were more likely to be assigned to a longer attempt of non-operative treatment resulting in a longer time-to-surgery. No physical follow-up examinations were performed for functional outcome assessment.

Still, we were able to assess the one-year mortality according to the time-to-surgery in all patients included in the study with a complete one-year follow-up of mortality data based on insurance data.

In summary, mortality and in-hospital complications remain high among geriatric patients with osteoporotic vertebral fractures when treated operatively. Based on the results of our retrospective study, one could hypothesize that time-to-surgery is not a factor that can change mortality rates or in hospital complication rates.

Treatment decisions for these fractures in the elderly depend on multiple factors and should be individualized. The findings of this study may help surgeons plan surgical intervention in more detail and may allow surgeons to avoid stabilizing these fractures within the first 48 h after diagnosis.

## Conclusions

In contrast to patients with proximal femur factures, time-to-surgery had no significant effect on one-year mortality in geriatric patients with osteoporotic vertebral compression fractures. Treatment decisions for these fractures in the elderly should be individualized.”

## Abbreviations

ASA: American Society of Anesthesiologists Scale
SD: Standard deviation
VCF: Vertebral compression fracture
